# Reversal of Hartmann’s procedure: The impact of timing – a single-tertiary-center experience

**DOI:** 10.1016/j.sipas.2025.100292

**Published:** 2025-06-15

**Authors:** Sascha Vaghiri, Maria Chara Stylianidi, Laura Engelmann, Eslam Elmaghraby, Levent Dizdar, Wolfram Trudo Knoefel, Hermann Kessler, Dimitrios Prassas

**Affiliations:** a Department of Surgery (A), Heinrich-Heine-University, Medical Faculty and University Hospital Duesseldorf, Duesseldorf, Germany; b Medical Research School Duesseldorf, Heinrich-Heine-University Duesseldorf, Moorenstr. 5, 40225 Duesseldorf, Germany; c Department of Colorectal Surgery, Digestive Disease Institute, Cleveland Clinic, 9500 Euclid Avenue, Cleveland, Ohio 44195, USA; d Department of Surgery, Katholisches Klinikum Essen, Philippusstift, Teaching Hospital of Duisburg-Essen University, Huelsmannstrasse 17, 45355 Essen, Germany

**Keywords:** Hartmann’s reversal · Timing · Colorectal surgery · Morbidity

## Abstract

**Introduction:**

The optimal timing for Hartmann’s reversal remains a topic of ongoing debate. This study aimed to assess postoperative outcomes in patients undergoing early versus late Hartmann’s reversal at a tertiary academic center in Germany.

**Methods:**

A single-center retrospective cohort study was conducted, including all patients who underwent Hartmann’s reversal between January 2008 and July 2020. Patients were stratified into early (ER) and late (LR) reversal groups using a median cut-off value of 159 days. Operative outcomes including major morbidity and overall postoperative complications were compared between both groups. Factors associated with major postoperative morbidity were detected using uni- and multivariate regression models.

**Results:**

A total of 133 patients classified into the ER (n = 67, 50.38%) and LR (n = 66, 49.62%) groups were analyzed. There were no significant differences in overall morbidity (ER 56.72% versus LR 39.39%, p = 0.057) and major morbidity rates (Clavien-Dindo ≥ IIIa) (ER 28.36% versus LR 21.21%, p = 0.423) between both groups. On multivariate analysis, smoking (p = 0.006), chronic renal disease (p = 0.003) and anastomotic configuration (p = 0.003) were identified as significant factors contributing to major morbidity after Hartmann’s reversal.

**Conclusion:**

Hartmann’s reversal is still associated with an increased risk of postoperative complications. However, timing of Hartmann’s reversal does not seem to influence postoperative morbidity. Of note, patient-related modifiable factors as well as the anastomotic configuration are important determinants of major complication occurrence.

## Introduction

In 1921, French surgeon Henri Hartmann introduced a surgical technique for treating distal colon tumors, later known as the Hartmann procedure (HP), which gained widespread recognition in the 1970s [1,2]. This procedure involves the resection of the diseased segment of the left-sigmoid colon, diversion of the proximal colon to create a terminal colostomy, and closure of the rectal stump [2]. While it is rarely employed for elective colorectal cancer surgery today, the Hartmann procedure remains a vital, life-saving surgical technique in emergencies such as bowel obstruction, complicated diverticulitis, inflammatory colitis, volvulus, ischemia, or primary colonic anastomotic leaks [[2], [3], [4]] The restoration of bowel continuity, known as Hartmann's reversal, originally described in 1950 by Allen Boyden, should ideally follow the Hartmann procedure as a second operation [5]. Hartmann’s reversal is a technically complex procedure, primarily due to challenges such as adhesions, chronical pelvic infection, abdominal obesity, limited access to the rectal stump and short rectal stump [4,6]. Based on published literature, it is performed in only 35%–60% of patients after end colostomy repair, with morbidity rates ranging from 30%–60% and mortality rates reaching up to 7% [[6], [7], [8], [9], [10]]. Surprisingly, there is a significant lack of high-quality evidence in the current literature regarding factors influencing Hartmann's reversal, as evidenced by the lack of meta-analyses on this topic [11]. Consistent with this pattern, there are limited studies examining the optimal timing of Hartmann's reversal [2,8,[12], [13], [14], [15], [16], [17]]. Our study therefore aims to investigate the influence of the timing of Hartmann’s reversal on the postoperative course and the frequency of operative related complications. Furthermore, identifying factors associated with major morbidity was the secondary goal of the analysis.

## Materials and Methods

### Patient collective and study design

All patients who underwent a Hartmann's reversal procedure at the Department of General, Visceral, and Pediatric Surgery, Medical Faculty and University Hospital Düsseldorf, Germany, between January 2008 and July 2020, were identified from a large prospectively maintained database and consecutively included in this retrospective cohort study. All patients underwent a standardized preoperative evaluation and workup respectively, which included laboratory tests, contrast enema, digital rectal examination, bowel preparation and colonoscopy if one had not been performed previously. In cases of colorectal cancer, appropriate follow-up examinations were performed.

The majority of anastomoses were performed in an end-to-end fashion using a 31-mm manual circular stapler. In cases with an extended Hartmann stump, hand-sewn or stapled anastomoses were created. All Hartmann’s reversals were performed via laparotomy. The severity of postoperative morbidity was classified according to Clavien-Dindo [18]. Chronic kidney disease was defined based on the latest nephrological guidelines [19].

Standard postoperative care included routine laboratory monitoring, early mobilization and staged reintroduction of a normal diet after confirmed bowel movement. Patients with prolonged postoperative bowel paralysis were provided with supplemental total parenteral nutrition (TPN). After discharge from the hospital, all patients attended follow-up appointments at our outpatient clinic for clinical assessment.

This study was approved by the Institutional Ethics Committee of the Medical Faculty at Heinrich-Heine-University, Duesseldorf, Germany (Study Number 2022-1855). It was conducted in compliance with the Strengthening the Reporting of Observational Studies in Epidemiology (STROBE) guidelines for observational research [20]. Informed consent was waived because no data regarding the cases were disclosed.

### Data collection and group definition

Medical and operative charts for each included patient were reviewed to collect the following parameters and information:1) demographics [age, gender, and body mass index (BMI)], American Society of Anesthesiologists (ASA) classification, comorbidities, immunosuppressive medication, 2) operative-specific details [type of index surgery, time point between index surgery and reversal, type of access, anastomotic configuration and technique, ostomy location, surgery duration (min), degree of adhesions as judged by the performing surgeons, concomitant hernia repair, volume substitution (ml)], 3) postoperative complications (impaired wound healing, burst abdomen, anastomotic leak, anastomotic bleeding, ileus, urinary tract infection, urinary leak, intraperitoneal abscess, intraperitoneal hematoma, major morbidity classified as Clavien-Dindo ≥ IIIa), overall morbidity and postoperative hospital stay (days). All patients included in the analysis were categorized into two groups—early reversal (ER) and late reversal (LR)—based on the timing of Hartmann's reversal using the median time interval (159 days) of the entire cohort between index surgery and the Hartmann’s reversal procedure. The primary outcome was the rate of major postoperative morbidity while all other postoperative complications were defined as the secondary points of interest.

### Statistical analysis

Statistical analyses were conducted using SPSS software version 23.0 (IBM Corp., Armonk, NY). Continuous variables were reported as mean ± standard deviation (SD) and assessed for normality prior to comparison. For normally distributed data, the t-test was applied, while the Mann-Whitney U test was used for non-normally distributed data. Categorical variables were expressed as frequencies (%) and compared using Fisher's exact test or the chi-squared test as appropriate.

To identify risk factors for major morbidity (Clavien-Dindo ≥ IIIa), uni- and multivariate analysis were performed. Variables with a p-value of < 0.05 were entered in a multivariable logistic regression model. Hazard ratios (HRs) with 95% confidence intervals (CIs) were estimated. A p-value of < 0.05 was considered statistically significant for all analyses.

## Results

### Patient characteristics

During the study period, 133 patients underwent Hartmann’s reversal at our department. Using a cutoff of 159 days (range 4-867 days) between the index operation and the ostomy reversal procedure, 67 patients (50.38%) were assigned to the ER group, while 66 patients (49.62%) were included in the LR group. Patient characteristics are summarised in Table 1. The cohort included 72 men (54.14%) and 61 women (45.86%), with a mean age of 60.76 ± 12.76 years in the ER group and 63.32 ± 14.71 years in the LR group at the time of reversal surgery. Baseline demographics including sex, age, BMI and ASA score were not significantly different between the two groups. Comorbidities including diabetes, arterial hypertension, chronic renal failure, and pulmonary and cardiac diseases were also equally distributed between both cohorts.

**Table 1:** Patient characteristics by reversal timing.

	ER (n = 67)	LR (n = 66)	P-Value
Gender, (n; %)			0.863
Male	37 (55.22)	35(53.03)	
Female	30(44.77)	31 (46.97)	
Age, (mean ± SD)	60.76 ± 12.76	63.32 ± 14.71	0.286
BMI, (mean ± SD)	22.5 ± 4.94	23.22 ± 5.85	0.449
ASA, (n;%)			0.248
- I - II - III - IV	5 (39.51) 37 (55.22) 25 (37.31) 0 (0)	6 (9.09) 33 (50) 23 (34.85) 4 (6.06)	
Diabetes, (n; %)	4 (5.97)	8 (12.12)	0.242
Hypertension, (n; %)	38 (56.72)	48 (72.72)	0.07
Chronic renal failure, (n; %)	10 (14.93)	10 (15.15)	1
Immunosuppression, (n; %)	9 (13.43)	7 (10.60)	0.791
Pulmonary disease, (n; %)	13 (19.40)	16 (24.24)	0.532
Cardiovascular disease, (n; %)	26 (38.80)	25 (37.87)	1
Smoking, (n; %)	12 (17.91)	12 (18.18)	1

SD: Standard Deviation, BMI: body mass index, ASA: American Society of Anesthesiologists

### Operative characteristics and morbidity of the initial Hartmann procedure

Sigmoid diverticulitis was the predominant indication for the initial Hartmann procedure in both groups (ER: 87.05% versus LR: 68.18%), as shown in Table 2. The late reversal group had a slightly higher proportion of colorectal cancer patients (ER: 4.47% versus LR: 18.18 %) (Table 2). The majority of cases at the index surgery were performed as emergency procedures (ER: 97.01% versus LR: 90.9%) through laparotomy. The predominant localization of the end ostomy was the descending colon (ER: 88.06% versus LR: 83.33 %) followed by the transverse colon (ER: 11.94% versus LR: 16.67%). The duration of surgery was longer in the late reversal group (ER: 173 ± 57.21 min versus LR: 192.74 ± 75.68 min) although not statistically significant (p = 0.119). Stoma-related morbidity (ER: 19.4% versus LR: 27.27%) and parastomal hernia rates (ER: 12.9% versus LR: 16.67%), were non-significantly higher in the LR group.

**Table 2:** Index surgery characteristics.

	ER (n = 67)	LR (n = 66)	P-Value
Primary Access, (n; %)			0.441
- Laparoscopic - Open	2 (2.99) 65 (97.01)	4 (6.06) 62 (93.93)	
Conversion, (n; %)	1 (1.49)	4 (6.06)	0.208
Index surgery indication, (n; %)			0.062
- Malignancy - Sigmoid diverticulitis - Colonic ischemia - Other	3 (4.47) 57 (85.07) 4 (5.97) 3 (4.47)	12 (18.18) 45 (68.18) 5 (7.58) 4 (6.06)	
Surgery duration, (mean ± SD)	173 ± 57.21	192.74 ± 75.68	0.119
Emergency surgery, (n; %)	65 (97.01)	60 (90.9)	0.165
Ostomy localization, (n; %)			0.468
- Transverse colon - Descending colon	8 (11.94) 59 (88.06)	11 (16.67) 55 (83.33)	
Parastomal hernia, (n; %)	8 (12.9)	11 (16.67)	0.110
Stoma associated morbidity, (n; %)	13 (19.4)	18 (27.27)	0.311

### Operative characteristics and course

In both groups, all reversal procedures were performed through laparotomy. The majority of anastomoses were created with staplers (ER: 86.57% versus LR: 93.93%). Concomitant hernia repair by means of direct fascia closure, was significantly more common in the LR group (ER: 16.42% versus LR: 31.81%, p = 0.044). Additionally, the intraoperative crystalloid volume substitution was significantly higher in the LR group (ER: 4433 ± 2469 ml versus LR: 5946 ± 2792 ml, p = 0.003).

### Postoperative Complications

Postoperative morbidity was observed in 64 patients (48.12%) of the entire study cohort. The incidence of impaired wound healing was significantly greater in the ER group (ER: 46.26% versus LR: 22.73%, p = 0.004) (Table 3). Overall morbidity (p = 0.057) and major morbidity (Clavien-Dindo ≥ IIIa) (p = 0.423) did not differ significantly between the two groups. Moreover, surgery duration (p = 0.093), postoperative hospital stay (p = 0.921), burst abdomen (p = 0.347), anastomotic leak (p = 0.441), anastomotic bleeding (p = 0.748), ileus (p = 0.680), urinary tract infection (p = 0.619), urinary leak (p = 1), intraperitoneal abscess (p = 0.441), and intraperitoneal hematoma (p = 0.244) showed no significant differences when comparing ER and LR groups (Table 3). Univariate analysis revealed ASA score, smoking, chronic renal disease, and anastomotic configuration as significant determinants of major postoperative morbidity (Clavien-Dindo ≥ IIIa) with a p value < 0.05. Upon multivariate analysis of the above-mentioned variables, chronic renal disease (OR 5.030 95% CI (1.719-14.720), p = 0.003), smoking (OR 4.213 95% CI (1.520-11.678), p = 0.006) and anastomotic configuration (OR 6.728, 95% CI (1.719-14.720), p = 0.003) were found to be independent predictive factors for major postoperative morbidity (Clavien-Dindo ≥ IIIa). The cumulative results of uni- and multivariate analysis are presented in Table 4.

**Table 3:** Reversal surgery characteristics and postoperative outcomes.

	ER (n = 67)	LR (n = 66)	P-Value
Anastomotic technique, (n; %)			0.242
- Stapler - Hand-sewn	58 (86.57) 9 (13.43)	62 (93.93) 4 (6.06)	
Concomitant hernia repair, (n; %)	11 (16.42)	21 (31.81)	**0.044**
Surgery duration, (mean ± SD)	220.10 ± 71.75	246.77 ± 106.25	0.093
Volume substitution, (mean ± SD)	4433 ± 2469	5946 ± 2792	**0.003**
In-hospital stay, (mean ± SD)	16.76 ± 15	17.05 ± 18.08	0.921
Impaired wound healing, (n; %)	31 (46.26)	15 (22.73)	0.004
Burst abdomen, (n; %)	4 (5.97)	2 (3.03)	0.347
Anastomotic leak, (n; %)	5 (7.46)	2 (3.03)	0.441
Anastomotic bleeding, (n; %)	1 (1.49)	1 (1.51)	0.748
Ileus, (n; %)	4 (5.97)	2 (3.03)	0.680
Urinary tract infection, (n; %)	1 (1.49)	2 (3.03)	0.619
Intraperitoneal abscess, (n; %)	2 (2.99)	4 (6.06)	0.441
Intraperitoneal hematoma, (n; %)	0 (0)	2 (3.03)	0.244
Urinary leak, (n; %)	1 (1.49)	0 (0)	1
Clavien-Dindo ≥ IIIa, (n; %)	19 (28.36)	14 (21.21)	0.423
Overall morbidity, (n; %)	38 (56.72)	26 (39.39)	0.057

**Table 4:** Uni- and multivariate analysis of factors affecting major morbidity (CD ≥ IIIa).

	CD < IIIa (n = 100)	CD ≥ IIIa (n = 33)	P-Value	Odds ratio (95% CI)	P-Value
	***Univariate Analysis***			***Multivariate Analysis***	
Timing of reversal, (n; %)			0.423		
- Early	48/67 (71.64)	19/67 (28.36)			
- Late	52/66 (78.79)	14/66 (21.21)			
Gender, (n; %) - Male - Female	53/72 (73.61) 47/61 (77.05)	19/72 (26.39) 14/61 (22.95)	0.691		
Age, (mean ± SD)	61.17 ± 12.76	64.64 ± 12.8	0.193		
BMI, (mean ± SD)	23.03 ± 5.67	22.34 ± 4.55	0.490		
ASA, (n; %)			**0.020**		
- I - II - III - IV	11/11 (100) 56/70 (80) 31/48 (64.58) 2/4 (50)	0 14/70 (20) 17/48 (35.42) 2/4 (50)			
Diabetes, (n; %) - Yes - No	8/12 (66.66) 92/121 (76.03)	4/12 (33.33) 29/121 (23.97)	0.491		
Hypertension, (n; %) - Yes - No	67/86 (77.9) 33/47 (70.2)	19/86 (22.1) 14/47 (29.8)	0.402		
Chronic renal disease, (n; %) - Yes - No	9/20 (45) 91/113 (80.53)	11/20 (55) 22/113 (19.47)	0.002	**5.030** **(1.719-14.720)**	**0.003**
Immunosuppression, (n; %) - Yes - No	10/16 (62.5) 90/117 (76.92)	6/16 (37.5) 27/117 (23.08)	0.171		
Pulmonary disease, (n; %) - Yes - No	22/29 (75.86) 78/104 (75)	7/29 (24.14) 26/104 (25)	1		
Cardiovascular disease, (n; %) - Yes - No	36/51 (70.59) 64/82 (78.05)	15/51 (29.41) 18/82 (21.95)	0.410		
Smoking, (n; %) - Yes - No	13/24 (54.17) 87/109 (79.81)	11/24 (45.83) 22/109 (20.18)	0.017	**4.213** **(1.520-11.678)**	**0.006**
Index surgery indication, (n; %) - Malignancy - Sigmoid diverticulitis - Colonic ischemia - Other	10/15 (66.66) 76/102 (74.51) 8/9 (88.89) 6/7 (85.71)	5/15 (33.33) 26/102 (25.49) 1/9 (11.11) 1/7 (14.29)	0.663		
Index surgery emergency, (n; %) - Yes - No	94/125 (75.2) 6/8 (75)	31/125 (24.8) 2/8 (25)	1		
Ostomy localization, (n; %) - Transverse colon - Descending colon	11/19 (57.86) 89/114 (78.07)	8/19 (42.11) 25/114 (21.93)	0.083		
Anastomotic configuration, (n; %) - End-to-end - Other	94/119 (78.99) 6/14 (42.86)	25/119 (21.01) 8/14 (57.14)	0.006	**6.728** **(1.941-23.322)**	**0.003**
Anastomotic technique, (n; %) - Stapler - Hand-sewn	88/120 (73.33) 12/13 (92.31)	32/120 (26.66) 1/13 (7.69)	0.184		
Adhesions at reversal, (n; %) - None - Mild - Moderate - Dense	1/2(50) 6/11 (54.54) 28/39 (71.79) 65/81 (80.24)	1/2(50) 5/11 (45.45) 11/39 (28.21) 16/81 (19.76)	0.167		
Volume substitution at reversal, (mean ± SD)	5329.27 ± 267	4851.85 ± 293	0.458		
Surgery duration at reversal, (mean ± SD)	234.07 ± 89.56	231.12 ± 97.3	0.878		

CD: Clavien-Dindo, CI: Confidence Interval, BMI: Body mass index, ASA: American Society of Anesthesiologists, SD: Standard Deviation

## Discussion

We conducted a retrospective study to assess the impact of timing on intraoperative and postoperative outcomes following Hartmann’s reversal. Our analysis of 133 patients is among the largest published on this topic. Using a 159-day cut-off time interval, we found no significant difference in overall and major morbidity between early and late reversal groups. However, multivariate analysis identified smoking status, chronic renal disease, and non-end-to-end anastomotic configuration as predictors of major morbidity (Clavien-Dindo ≥ IIIa).

Although surgical techniques have advanced, Hartmann's reversal remains a highly complex procedure with significant postoperative morbidity and mortality. According to published literature, ostomy reversal is performed in fewer than 50% of patients who have undergone a Hartmann’s procedure [4,21,22]. Several factors, including age, gender, malignancy, comorbidities, ASA score, immunosuppression, and chemotherapy, are carefully considered before proceeding with the reversal [[23], [24], [25]]. The optimal timing for Hartmann's reversal remains a topic of debate, with published studies yielding conflicting results and the average interval being approximately seven to eight months from the index surgery to ostomy reversal [11,16,26].

The first study on this subject by Roe et al. [14], which included 69 patients, found no increase in morbidity when colostomy closure was performed before four months or even three months. Based on these findings, early restoration of bowel continuity is recommended when the rectal stump has favourable accessibility [14]. The concern that delayed Hartmann's reversal may be associated with rectal stump atrophy, making intraoperative identification more difficult and leading to more complications, is supported by the findings of Roque-Castellano et al. and Tan et al. [27,28].

Keck et al. [12]. performed a retrospective analysis of 50 patients, drawing similar conclusions to Roe et al. regarding overall morbidity [12,14] . Patients were divided into two cohorts: early reversal (< 4 months, n = 13) and late reversal (> 4 months, n =3 7). The study found no significant differences in mortality, overall morbidity, or anastomotic leak rates between the groups. However, the median hospital stay was longer in the early reversal group (17 days versus 12 days), and the mean adhesion density grade was higher (2.6 versus 2.1). Furthermore, intraoperative small bowel injury occurred more frequently in the early reversal group.

Pearce et al. [13] conducted a retrospective analysis of 80 patients, stratified into two cohorts: early reversal (< 6 months) and late reversal (> 6 months). Their findings indicated that early reversal was associated with increased morbidity, prolonged hospital stay, and higher mortality. The observed increase in morbidity within the early reversal group was attributed to the patients' poorer overall condition, as they had insufficient time to recover from the initial surgery. Additionally, the presence of intra-abdominal sepsis and ongoing inflammation further contributed to the heightened risk profile in this cohort [13]. A study by Banerjee et al. [29]. supports the same thesis, indicating that a longer waiting interval before Hartmann's reversal has been shown to improve the clinical and nutritional status of patients and promote resolution of the underlying pathology [29].

These studies were carried out in the 1990s. More recent studies on the timing of Hartmann's reversal have been performed by Flemming et al. [8], Horesh et al. [16], Clementi et al. [2] and Popazu et al. [30]. Flemming and colleagues analyzed 76 patients who underwent Hartmann's reversal for diverticular perforation and assessed postoperative complications in relation to timing [2]. Their results suggested that a longer interval between the initial procedure and reversal was associated with an increased risk of postoperative complications. These results contrast with those of Horesh et al. [16], who studied 122 patients and found no significant correlation between timing of reversal and postoperative outcomes. Popazu et al. investigated the optimal timing for Hartmann’s reversal in patients who had undergone the procedure for sigmoid diverticulitis. Their analysis demonstrated that colostomy reversal performed within 45 to 120 days postoperatively was associated with improved outcomes, including lower rates of complications—such as wound infections and postoperative abscesses—and a shorter hospital stay. The authors emphasized that timing should be individualized based on each patient’s clinical status, with early reversal favored in those without significant comorbidities [30]. In a further study of 105 patients, Clementi et al. [2] concluded that early Hartmann's reversal was associated with a significantly lower complication rate. However, they also observed a higher incidence of incisional ventral hernia in the early reversal group [2].

The largest prognostic cohort study to date, conducted by Resio et al. [17] from the United States with 1660 patients, found no significant differences in mortality, transfusion rates, ileus, or major complications among patients undergoing Hartmann’s reversal within one year. However, earlier reversal was associated with a shorter length of stay and fewer hospital readmissions [17].

Our study identified smoking, chronic kidney disease and non-end-to-end anastomotic configuration as predictors of major morbidity after Hartmann's reversal. The impact of smoking and chronic kidney disease on postoperative complications is consistent with findings in the published literature. A meta-analysis of 107 studies by Grønkjær et al. demonstrated that smoking increases the risk of complications after major colorectal surgery, with current smokers exhibiting the highest risk [31]. Similarly, a meta-analysis by Mills et al. (2011) [32] and a retrospective study by Inoue et al.[33] found that smoking cessation was associated with reduced postoperative complications and shorter hospital stays following abdominal surgery [32,33]. Regarding chronic kidney disease, multiple studies have shown its association with higher morbidity in both abdominal and colorectal surgeries [[34], [35], [36]].

Our findings indicate that the anastomotic configuration significantly influences the incidence of major postoperative complications. Among the techniques analyzed, end-to-end anastomosis demonstrated the lowest rate of Clavien-Dindo ≥ 3 complications. However, the overall anastomotic-related morbidity in our cohort remained low (leakage: 5.26%, bleeding: 1.50%), limiting the ability to perform an in-depth analysis of contributing factors. These results are consistent with the meta-analysis by McKechnie et al. [37], which included six randomized controlled trials comprising 270 patients. Their findings suggest that end-to-end anastomosis reduces the risk of anastomotic leakage in patients undergoing low anterior resection [37]. A retrospective study by Chierici et al. [38], analyzing 518 patients who underwent laparoscopic rectal cancer surgery for mid and lower rectal tumors, reported a lower rate of radiologic anastomotic leakage in end-to-end anastomosis compared to end-to-side anastomosis. However, no significant difference was observed in terms of clinical leakage [38]. Conversely, a larger retrospective study by Alahmadi et al. [39], which included 844 patients undergoing left-sided colonic and rectal resections for colorectal cancer, found no significant difference in clinical anastomotic leakage between different anastomotic techniques [39]. These findings highlight the need for further high-quality studies to clarify the impact of anastomotic configuration on postoperative outcomes.

The ostomy reversal timing is predominantly influenced by patient-specific factors and intraoperative variables rather than by differences in postoperative morbidity and mortality. Nonetheless, the psychological distress and mental health challenges associated with living with an ostomy are critical considerations, as ostomy reversal can markedly enhance overall well-being. In our study, stoma-associated morbidity was observed in 31 patients (23.31%). In such cases, early Hartmann’s reversal should be considered, given the non-significant differences between early and late reversal.

Our study reveals a higher wound infection rate in patients who underwent early Hartmann's reversal. While existing studies acknowledge that wound infection is a common complication with a higher incidence than in elective surgery, they do not specifically assess whether early reversal increases this risk [2,8,14]. A possible explanation could be the poorer general condition of these patients, along with intra-abdominal sepsis and ongoing inflammation, as early reversal allows patients less recovery time compared to late reversal [13].

An important limitation of our observational study is that the decision to perform Hartmann’s reversal was largely influenced by factors such as surgeon clinical judgement and individual patient characteristics, which may introduce selection bias and affect the interpretation of our results. In addition, the relatively small sample sizes of both the ER and LR cohorts, combined with the low rate of anastomotic-related complications, may limit adequate statistical power for analysis and conclusions. Randomised controlled trials with standardised surgical protocols are needed to establish the optimal timing for Hartmann's reversal. The results of such studies should be incorporated into future national and international practice guidelines to ensure evidence-based decision making.

## Conclusions

The timing of Hartmann's reversal did not influence operative outcomes. Our study identified modifiable patient-related and technical factors that contribute to major complications. Therefore, emphasis should be placed on addressing these modifiable factors. However, as the current evidence is largely based on retrospective studies, prospective studies are needed.

## CRediT authorship contribution statement

**Sascha Vaghiri:** Writing – original draft, Methodology, Formal analysis, Data curation, Conceptualization. **Maria Chara Stylianidi:** Writing – original draft, Investigation, Formal analysis. **Laura Engelmann:** Data curation. **Eslam Elmaghraby:** Data curation. **Levent Dizdar:** Writing – review & editing. **Wolfram Trudo Knoefel:** Writing – review & editing. **Hermann Kessler:** Writing – review & editing. **Dimitrios Prassas:** Methodology, Formal analysis, Conceptualization.

## Declaration of competing interest

The authors declare that they have no known competing financial interests or personal relationships that could have appeared to influence the work reported in this paper.
